# Left bundle branch pacing in third-degree atrioventricular block following morrow surgery: a case report

**DOI:** 10.3389/fcvm.2024.1391047

**Published:** 2024-07-26

**Authors:** Keqiang Huang, Hongmei Gan, Jingjing Jiang, Cheng Tang

**Affiliations:** Department of Cardiology, Wuhan Asia General Hospital, Wuhan, Hubei, China

**Keywords:** left bundle area pacing, left bundle block, morrow, septal myectomy, pacemaker

## Abstract

Left bundle branch pacing (LBBP) has proven to be an alternative method for delivering physiological pacing to achieve electrical synchrony of the left ventricle (LV), especially in patients with atrioventricular block and left bundle branch block (LBBB). However, it is unclear whether it still achieved in patients whose left bundle branch (LBB) has had surgery-induced damage. The Morrow operation (Morrow septal myectomy) is regarded as one of the most effective treatments for hypertrophic obstructive cardiomyopathy (HOCM). The surgery resects small sections of muscle tissue in the proximal ventricular septum nearby or contains the LBB, which means that physical damage to the LBB is almost inevitable. Approximately 2%–12% of patients may need pacemaker implanted after Morrow surgery. LBBP is a feasible and effective method for achieving electric resynchronization of LBBB compared to right ventricular pacing (RVB). Nevertheless, there is a dearth of data on LBBP in third-degree atrioventricular block (AVB) following Morrow surgery. We report a case of successful LBBP in those patients.

## Introduction

Left bundle branch pacing (LBBP) has proven to be an alternative method for delivering physiological pacing to achieve electrical synchrony of the left ventricle (LV), especially in patients with atrioventricular block and left bundle branch block (LBBB) (1). However, it is unclear whether it still achieved in patients whose left bundle branch (LBB) has had surgery-induced damage.

The Morrow operation (Morrow septal myectomy) is regarded as one of the most effective treatments for hypertrophic obstructive cardiomyopathy (HOCM). The surgery resects small sections of muscle tissue in the proximal ventricular septum nearby or contains the LBB, which means that physical damage to the LBB is almost inevitable (2). Approximately 2%–12% of patients may need pacemaker implanted after Morrow surgery (3, 4).

LBBP is a feasible and effective method for achieving electric resynchronization of LBBB compared to right ventricular pacing (RVB). Nevertheless, there is a dearth of data on LBBP in third-degree atrioventricular block (AVB) following Morrow surgery. We report a case of successful LBBP in those patients.

## Key teaching points

• LBBP was achieved in the patient who received LBB and third-degree AVB following Morrow surgery.
• LBB is an area of the left ventricular septum instead of an electric wire, so even if the surgery physically damages it, it is not possible to affect all the electrical conduction characteristics of LBB.
• Physically damaged LBBB cannot be corrected by His-bundle pacing (HBP). Usually, no LBB potential can be recorded.
• The ECG characteristics [paced QRS morphology, paced QRS duration and stimulus to peak left ventricular activation time (Sti-LVAT)] could be evidence of LBB capture.a

## Case report

**Figure 1:**
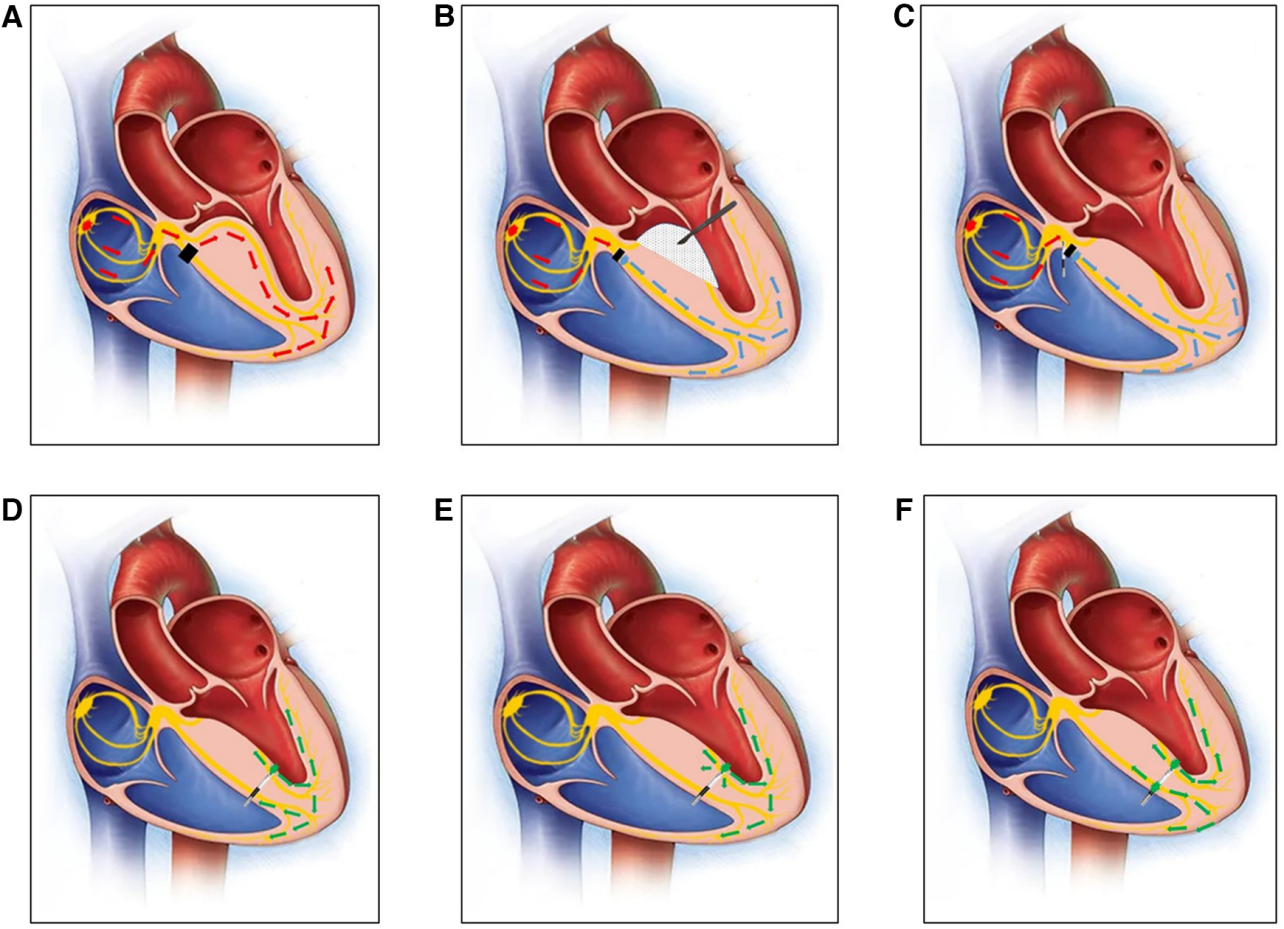
(**A**) A diagram of the right bundle branch block before MORROW surgery. The black square indicates that there was a block in the right bundle branch. (**B**) A diagram of how the left bundle branch has been damaged by the scalpel in MORROW surgery. The rhythm of the ventricle comes from under the block site of the RBB (the blue arrow). (**C**) A diagram of the His bundle potential recorded by the first 3830 lead. (**D**) A diagram of the 3830 pacing lead advanced from the right ventricular septum (RVS) to the left ventricular septum (LVS) in the subendocardium, and the lead captured the LBB under the damaged part caused by MORROW surgery (the green arrow). (**E**) A diagram of 3830 pacing lead capturing the LBB and the myocardium at the same time (the green arrow). (**F**) A diagram of 3830 pacing lead capturing the LBB and the right ventricular myocardium by the ring at the same time (the green arrow) after planting the permanent pacemaker.

**Figure 2:**
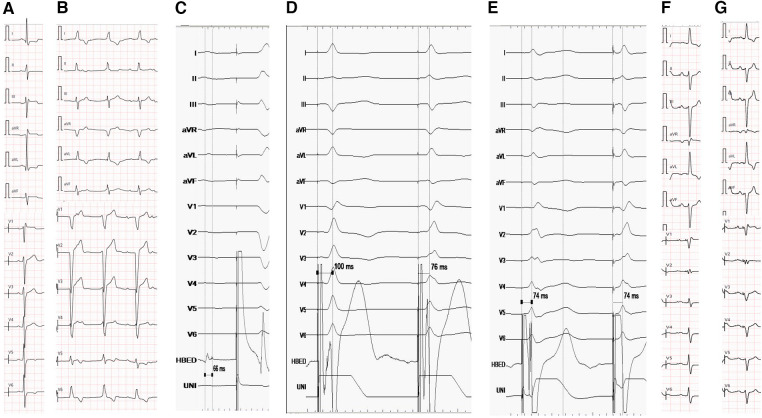
(**A**) ECG before MORROW surgery. ECG showed sinus bradycardia, RBBB and left ventricular hypertrophy (QRS duration:126 ms). (**B**) ECG showed 3rd degree AVB 7 days after MORROW surgery(QRS duration:130 ms). (**C**) The His bundle potential follows behind the atrial potential, but it cannot capture the ventricle activation or LBB even at high voltage (10 v/1 ms). (**D**) There was an abrupt shortening of Sti-LVAT from 100 ms to 76 ms while the pacing lead advanced from the RVS to the LVS in the subendocardium. (**E**) Sti-LVAT remains the same at low and high voltages (2 V and 10 V). (**F**) The ECG of the pacemaker, the morphology of the premature ventricle was the same as before the pacemaker was implanted(QRS duration:114 ms). (**G**) Electrocardiogram at the 6-month follow-up after pacemaker implantation.

A 55-year-old woman presented with symptoms of chest distress and syncope for 10 years. Ecg showed sinus bradycardia, right bundle branch block (RBBB) and left ventricular hypertrophy (Figures 1A, 2A). Echocardiography examination revealed the septum below the aortic valve; ventricular septal hypertrophy with left ventricular outflow tract stenosis; mild aortic stenosis and severe regurgitation; and enlargement of the ascending aorta.

A Bentall + Subaortic septum resection + Morrow surgery was performed, and a transepicardial temporary pacing lead was implanted when the ECG monitor showed complete atrioventricular block (AVB) after the heart resumes beating.

After 7 days of observation, ECG still showed 3rd degree AVB (Figures 1B, 2B), so the patient was indicated for permanent dual-chamber pacemaker implantation, and LBBP was performed. The pace lead (Model 3830; SelectSecure, Medtronic, Minneapolis, MN) was successfully implanted, and both paced and intrinsic intracardiac EGM and ECG were continuously recorded while the pacing lead advanced from the right ventricular septum (RVS) to the left ventricular septum (LVS) in the subendocardium, with a unipolar pacing output of 2 V/0.5 ms.

After locating the tricuspid valve annulus (TVA) and tricuspid septal leaflet by right ventriculography (Supplementary Video S1), it was easy to identify the HBP site and locate the His bundle potential following the atrial potential, but it could not capture the ventricular activation or LBB even at high voltage (10 v/1 ms) (Figures 1C, 2C).

A second 3830 lead was implanted to LBBP, and there was an abrupt shortening of Sti-LVAT from 100 ms to 76 ms while the pacing lead advanced from the RVS to the LVS in the subendocardium with a unipolar pacing output of 2 V/0.5 ms (Figures 1D, 2D). The Sti-LVAT remains the same at low and high voltages (2 V and 10 V) (Figures 1E, 2E). However, no LBB potential was recorded even with HBP at a high voltage (10 V).

The depth of lead insertion was approximately 1.3–1.5 cm by angiography through the C315 sheath (Supplementary Video S2). The QRS was narrow (124 ms) after implanting the permanent pacemaker, but there was frequent monomorphous ventricular premature beats, and the morphology of the premature ventricle was the same as before the pacemaker was implanted (Figures 1F, 2F).

## Discussion

Many studies have demonstrated that LBBP is feasible in LBBB patients and that the LBB potential could be recorded during His bundle pacing. However, whether it still works in patients whose LBB has suffered physical damage as a result of surgery, such as MORROW, has not been in-depth coverage. Past studies have mentioned that pacing of the conduction system is feasible in patients with hypertrophic cardiomyopathy. Jing-Jing and her colleagues' research (5) has demonstrated that CSP was safe and feasible in patients with HCM and cardiac dysfunction, and did not worsen cardiac performance especially in patients with LVEF <50%. HBP might be an effective alternative to LBBP in patients with significantly thickened interventricular septum. But in our case, the physical resection of the left bundle branch made His-bundle pacing unfeasible.

In this patient, RBBB existed before MORROW surgery, and a 3rd degree AVB was inevitably following the surgery, which almost certainly damaged the LBB. During pacemaker implantation, the His bundle potential was recorded behind the atrial potential (Figure 2C), which means that the block site was under the His bundle. HBP was not able to capture the ventricle activation or LBB even at high voltage (10 V/1 ms), indicating that HBP may not be effective in this kind of patient. Additionally, we supposed that it still did not work even if the patient had no RBBB before the surgery because the path from the His bundle to the LBB was damaged by the surgery.

Although the LBB potential could not be recorded after the physical damage caused by the surgery, there are various alternatives to confirm LBBP, including monitoring the paced QRS morphology when the mid notch of the QRS complex moves up and toward the end in lead V1. The paced ECG QRS morphology frequently presents as RBBB morphology with a low threshold. More direct evidence comes from the abrupt shortening of Sti-LVAT as the pacing lead advanced from the RVS to the LVS in the subendocardium with a unipolar pacing output of 2 V/0.5 ms (Figure 2D). More importantly, thanks to the help of John Jiang's connecting cable which consists of a rotatable port and a connection wire (6). We could continuously monitor and test during the procedure. Finally, LVAT remained the same at low- and high-output pacing. It also confirmed that the final implantation site of the LBBP was adjacent to the left conduction system (7).

In conclusion, LBBP could be obtained in patients who received LBB caused by physical damage from surgery, such as the Morrow surgery. There are various techniques to confirm LBBP. Further data are required to confirm whether it works in all of these kinds of patients.

## Data Availability

The original contributions presented in the study are included in the article/Supplementary Material, further inquiries can be directed to the corresponding author.
